# Editorial: Compassion: from neuroscience to new horizons and innovative, inclusive research agendas

**DOI:** 10.3389/fpsyg.2023.1324381

**Published:** 2023-11-06

**Authors:** Kathryn Waddington, Julian Manley, Trudi Edginton, Jason Kanov

**Affiliations:** ^1^School of Social Sciences, University of Westminster, London, United Kingdom; ^2^School of Health, Social Work and Sport, University of Central Lancashire, Preston, United Kingdom; ^3^School of Health and Psychological Sciences, City, University of London, London, United Kingdom; ^4^College of Business and Economics, Western Washington University, Bellingham, WA, United States

**Keywords:** compassion, neuroscience, organizations, leadership, ethic of care, health psychology, compassionate pedagogy

This Research Topic issue follows on from “Expanding the Science of Compassion” (Mongrain et al., 2021), which explored the neuroscience, physiological, psychological, and environmental aspects of the experience of compassion. This issue extends the understanding of compassion to include compassion in health psychology, pedagogical practice in higher education, organizations and leadership. It introduces innovative approaches from scholars working in diverse research contexts that include South Africa, Sri Lanka, and Slovenia, and highlights new horizons for organizational neuroscience research.

## Compassion in health psychology

Sandham and Deacon undertook a rapid review (16 articles) into the role of self-compassion into diabetes and its management. Their main findings were that self-compassion is associated with improved health outcomes (psychologically and medically) and can be enhanced through interventions such as the mindful self-compassion program. Self-compassion also features as a key factor in Halamová et al.'s analysis of best coping practices during the COVID-19 pandemic. Their study used the COPE Inventory to measure functioning coping behaviors with 1,683 Slovak nationals, followed by qualitative interviews with a randomly selected sample of nine participants with highest scores on all 15 subscales. Adaptive coping strategies were categorized into four domains, with self-compassion most frequently mentioned and elaborated upon, followed by compassion to *and* from others, and mutual compassion or “suffering together”. These two studies highlight the relationship between self-compassion, self-compassion training/interventions and well-being, which extends beyond health psychology to other areas of applied psychology. For example, see Kotera and Van Gordon's (2021) systematic review which demonstrates that self-compassion training can be effective in improving work-related well-being outcomes.

## Compassion in pedagogical practice

Jayasundara et al.'s mixed methods study used Gilbert's (2015) psychobiological model of compassion of: (i) drive; (ii) threat detection; and (iii) self-soothing, to inform a Cognitive Skills of Compassionate Communications (CSCC) training intervention for online group work meetings. Science, Technology, Engineering and Mathematics (STEM) students, from five Sri Lankan universities showed a significant improvement in screen-gaze attentiveness to one another, after the CSCC intervention. Lister et al. used a survey instrument co-designed with students with disclosed mental health conditions to examine barriers and enablers to student mental well-being in distance learning in a UK university. Assessment and life circumstances were the most significant barriers; enablers included curriculum and module content and building study skills. Students with disclosed mental health difficulties were consistently more likely to experience barriers, while enablers were experienced by all demographic groups. Hamilton and Petty's conceptual analysis of compassionate pedagogy for neurodiversity in higher education draws on principles of compassion-focused psychological therapies. Their article, framed within the neurodiversity paradigm, challenges pathologizing accounts of neurodevelopmental differences, including attention deficit disorder (ADHD), dyslexia, autism, developmental language disorder (DLD) and others. It considers how compassion can be enacted in curriculum design, interpersonal interactions, and leadership cultures in universities. A further two articles report original research into organizational compassion and leadership in universities (Denney; Pule and Gibney), reinforcing our argument that research into compassion must take place in compassionate institutional cultures.

## Compassion in organizations and leadership

Denney's qualitative study into how compassion was experienced in UK universities during COVID-19 also reveals the need for compassionate leadership cultures. A distinction is drawn between “formalized” compassion processes that prioritize compassion for students over that of staff, and “informal” compassion shown between staff. This study revealed failures in organizational compassion in higher education, with consequent staff suffering as a result of structurally embedding compassion for students. When seen alongside Jayasundara et al.'s work, these two studies emphasize the relevance of compassion for staff and student well-being in higher education organizational policy and practice (see also Waddington and Bonaparte, forth coming).

Pule and Gibney's qualitative study uses social dream drawing—a variation of the standard social dreaming approach—to investigate the quality of listening in the context of students and student leaders in South Africa. This study highlights the importance of inclusion of under-represented voices and psychosocial methods in organization compassion and leadership research. Their study shows how authentic and profound listening can lead to empathy; and through empathy, compassion. It is through deeply felt compassion that understanding emerges and provides a potential for “moving on”. Social dream drawing creates a safe, containing space for a heightened and “exquisite” empathic awareness for the exchange of areas of pain and trauma associated with colonialism and racism which otherwise would be too awful and unbearable for participants to share. The authors show how drawing on a tradition of investigating group psychodynamics—viewed from a socio-analytical or psychosocial lens—and an openness to a social or associative unconscious can provide a heretofore sparsely researched field in studies of compassion. The social dream drawing method as a process in itself is compassionate, in that judgements, solutions and answers are eschewed in favor of transparency and tolerance of all the contributions to the group process. Social dreaming has often been described as a “democratic” process; Pule and Gibney show how such a democracy in the fullness of its participatory quality can be a conductor of compassion.

Krause et al. qualitatively examine compassion within German work organizations in the context of dyads comprising leaders and their direct subordinates. Their analysis of interview data reveals paradoxes—opposing, interdependent tensions—which leaders and/or their subordinates may encounter during episodes of suffering and compassion. Their empirically grounded descriptions of several such paradoxes bring needed nuance to our understanding of challenges and opportunities associated with expressing suffering and enacting compassion in work settings. Their research also considers how social hierarchy influences the observed paradoxes, pointing to paradox navigation as a potentially crucial but under-appreciated (and under-researched) aspect of the competent practice of compassion, particularly for organizational leaders. More generally, this study's findings demonstrate the value of research that surfaces compassion recipients' and providers' experiences and that examines contextual influences impacting these experiences.

Spännäri et al. used adapted grounded theory methodology with nine focus group interviews that took place in Finland in private, public, and third sector organizations. Their study explored factors preventing and promoting innovation in organizations, asking more specifically: (i) how compassion is connected to these factors; and (ii) how compassion can boost innovation. Their analysis showed that innovation is profoundly and diversely interlinked with compassion, rather than being a single variable or practice. Existence of compassion promotes innovation, while absence/lack of compassion stifles innovation.

Of particular note is how the novel concept of *copassion*, defined as “the process of responding to the positive emotions of the other” (Pessi et al., 2022, p. 83), was found to be associated with innovation. While the term copassion is beginning to emerge into the lexicon of compassion studies, it is too early to say whether it is a potentially valuable future direction, or a wrong turn. Without further research, and conceptual and theoretical development it is too early to tell. However, the articles in this Research Topic issue provide material and methods to craft new and innovative interdisciplinary research agendas alongside more tried and tested approaches.

## New horizons in neuroscience

This Research Topic issue is positioned in the Frontiers in Organizational Psychology Speciality Section, and we were initially surprized at the lack of organizational neuroscience articles received. However, a number of relevant neuroscience focused studies featured in Mongrain et al.'s (2021) recent Research Topic, and a robust foundation for exploring the neuroscience of compassion has been well established (Seppällä et al., 2017). This provides converging theoretical, empirical, environmental and neuroscientific evidence that can be applied to a range of different settings at both individual and group levels of analysis. The intra and interpersonal nature of compassion toward self and others highlights the need for flexibility at the neurobiological level, observed within the parasympathetic nervous system to regulate heart rate variability (HRV) and vagal tone (Porges, 2017). These mechanisms underpin cognitive flexibility, emotional regulation, behavioral responses and interpersonal awareness that facilitate self-monitoring, self-soothing, empathy and response selection. The close alignment with the threat, drive, soothe processes within the psychobiological model of compassion (Gilbert, 2015) can inform understanding of individual, group and organizational processes. In turn, this can improve working relationships, compassionate leadership and promote the practice of kindness. While Jayasundara et al.'s article was the only one to explicitly refer to Gilbert's psychobiological model, neuroscience is implicit—if not cited—in the other articles in the issue.

The emerging field of Organizational Cognitive Neuroscience highlights the challenges, limitations and tensions that are associated with the complex imaging methods that are required to identify specific neurotransmitters and brain regions (Senior et al., 2011; Butler et al., 2016). In the field of organization and management research there is much to be gained in future collaborations between neuroscientists and scholars. For example, as we move toward more inclusive and more innovative research horizons the potential for technological advances in Immersive Virtual Reality settings, sensors, portable non-invasive imaging techniques and more extensive hormonal testing will provide greater opportunities to investigate the organizational neuroscience of compassion (Boukarras et al., 2021). The following key questions can be used to evaluate whether an empirical research program is worthwhile: (i) does it address a core problem in organizational/management research and/or practice? (ii) does it raise compelling new questions for neuroscientists? (iii) has it been neglected in other fields and is it likely to remain neglected? and (iv) will neural evidence add to our conceptual and theoretical understanding and, if so, how? (see Butler et al., 2016, p. 556).

In conclusion, this Research Topic demonstrates how qualitative and mixed methods research using innovative and inclusive approaches can shape future research agendas. It also draws attention to the need for compassionate cultures and leadership in universities—especially, we argue, in those that carry out research in these areas. This should further extend to all aspects of academic life and organization: compassion in the peer review processes for publication, research funding, appraisal systems, target setting, and expectations placed upon academics to produce impact at every step, even when impact is itself often hard to quantify and evidence.

Hoggett (2009) identified compassion as an essentially different quality to empathy, which he argues requires a degree of pity toward the object. The empathic process is a one-way process from subject to object and runs the danger of projecting unwanted and unnecessary thoughts and feeling into that other. Compassion, on the other hand, requires the participation of that other. Hoggett calls this “intelligent compassion whereby one can feel the pain and think critically about the injustice, thereby fusing an ethic of care to an ethic of justice” (2009, p. 147). Taken as a whole, the papers in this special edition provide foundations of a framework for a new structure of ethics formed through the fusion of care and justice, and we would like to thank the reviewers and Frontiers editorial team who have assisted us in this endeavor.

## Author contributions

KW: Writing—original draft, Writing—review & editing. JM: Writing—original draft, Writing—review & editing. TE: Writing—original draft, Writing—review & editing. JK: Writing—original draft, Writing—review & editing.
